# Systemic inflammation associates with and precedes cord atrophy in progressive multiple sclerosis

**DOI:** 10.1093/braincomms/fcae143

**Published:** 2024-04-20

**Authors:** Charlotte M Stuart, Aravinthan Varatharaj, Yukai Zou, Angela Darekar, Janine Domjan, Claudia A M Gandini Wheeler-Kingshott, V Hugh Perry, Ian Galea

**Affiliations:** Clinical Neurosciences, Clinical and Experimental Sciences, Faculty of Medicine, University of Southampton, Southampton SO16 6YD, UK; Clinical Neurosciences, Clinical and Experimental Sciences, Faculty of Medicine, University of Southampton, Southampton SO16 6YD, UK; Wessex Neurological Centre, University Hospital Southampton NHS Foundation Trust, Southampton SO16 6YD, UK; Clinical Neurosciences, Clinical and Experimental Sciences, Faculty of Medicine, University of Southampton, Southampton SO16 6YD, UK; Department of Medical Physics, University Hospital Southampton NHS Foundation Trust, Southampton SO16 6YD, UK; Department of Medical Physics, University Hospital Southampton NHS Foundation Trust, Southampton SO16 6YD, UK; Wessex Neurological Centre, University Hospital Southampton NHS Foundation Trust, Southampton SO16 6YD, UK; Department of Neuroinflammation, Faculty of Brain Sciences, NMR Research Unit, Queen Square Multiple Sclerosis Centre, UCL Queen Square Institute of Neurology, University College London, London WC1B 5EH, UK; School of Biological Sciences, University of Southampton, Southampton SO16 6YD, UK; Clinical Neurosciences, Clinical and Experimental Sciences, Faculty of Medicine, University of Southampton, Southampton SO16 6YD, UK; Wessex Neurological Centre, University Hospital Southampton NHS Foundation Trust, Southampton SO16 6YD, UK

**Keywords:** multiple sclerosis, progression, systemic inflammation

## Abstract

In preclinical models of multiple sclerosis, systemic inflammation has an impact on the compartmentalized inflammatory process within the central nervous system and results in axonal loss. It remains to be shown whether this is the case in humans, specifically whether systemic inflammation contributes to spinal cord or brain atrophy in multiple sclerosis. Hence, an observational longitudinal study was conducted to delineate the relationship between systemic inflammation and atrophy using magnetic resonance imaging: the SIMS (Systemic Inflammation in Multiple Sclerosis) study. Systemic inflammation and progression were assessed in people with progressive multiple sclerosis (*n* = 50) over two and a half years. Eligibility criteria included: (i) primary or secondary progressive multiple sclerosis; (ii) age ≤ 70; and (iii) Expanded Disability Status Scale ≤ 6.5. First morning urine was collected weekly to quantify systemic inflammation by measuring the urinary neopterin-to-creatinine ratio using a validated ultra-performance liquid chromatography mass spectrometry technique. The urinary neopterin-to-creatinine ratio temporal profile was characterized by short-term responses overlaid on a background level of inflammation, so these two distinct processes were considered as separate variables: background inflammation and inflammatory response. Participants underwent MRI at the start and end of the study, to measure cervical spinal cord and brain atrophy. Brain and cervical cord atrophy occurred on the study, but the most striking change was seen in the cervical spinal cord, in keeping with the corticospinal tract involvement that is typical of progressive disease. Systemic inflammation predicted cervical cord atrophy. An association with brain atrophy was not observed in this cohort. A time lag between systemic inflammation and cord atrophy was evident, suggesting but not proving causation. The association of the inflammatory response with cord atrophy depended on the level of background inflammation, in keeping with experimental data in preclinical models where the effects of a systemic inflammatory challenge on tissue injury depended on prior exposure to inflammation. A higher inflammatory response was associated with accelerated cord atrophy in the presence of background systemic inflammation below the median for the study population. Higher background inflammation, while associated with cervical cord atrophy itself, subdued the association of the inflammatory response with cord atrophy. Findings were robust to sensitivity analyses adjusting for potential confounders and excluding cases with new lesion formation. In conclusion, systemic inflammation associates with, and precedes, multiple sclerosis progression. Further work is needed to prove causation since targeting systemic inflammation may offer novel treatment strategies for slowing neurodegeneration in multiple sclerosis.

## Teaser

Systemic inflammation affects the central nervous system; whether it contributes to multiple sclerosis progression was unknown. Stuart *et al.* followed 50 people with progressive multiple sclerosis, measuring urinary neopterin weekly for 2.5 years. Systemic inflammation associated with and preceded MRI markers of progression, and may be a modifiable therapeutic target.

## Graphical Abstract

**Graphical Abstract:**
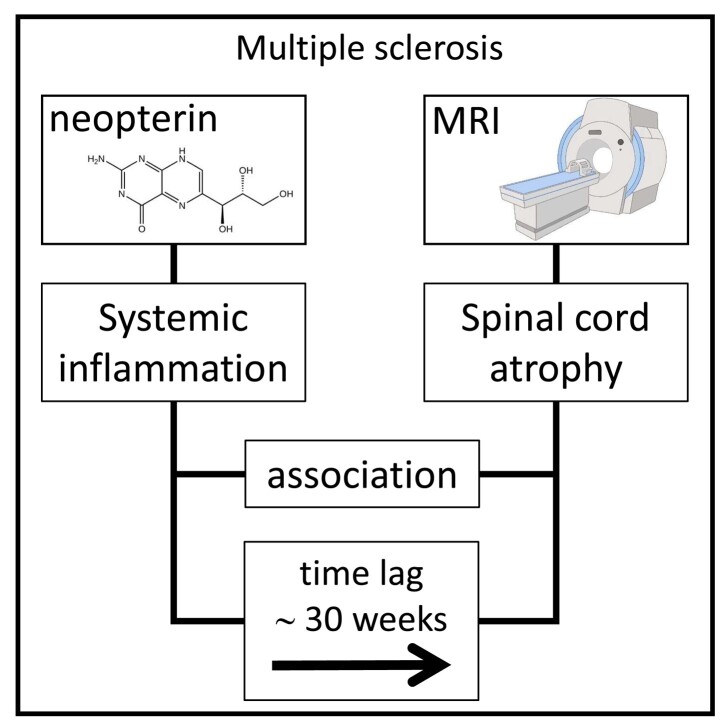

## Introduction

Multiple sclerosis (MS) is an autoimmune condition affecting the central nervous system (CNS) leading to a gradual progressive accumulation of irreversible neurological disability. Relapses occur in the early stages of the condition but progression is frequently dissociated from relapses. While treatments to prevent relapses are available, effective treatments to slow down or prevent progression are lacking. For this reason, progression has been identified as a priority for research.^1^ Axonal loss occurs in MS^2,3^ and there is robust evidence showing that the pathological substrate of progressive disability is axonal loss.^4-6^ Systemic inflammation, occurring outside the CNS, impacts on axonal loss through activation of the brain’s innate immune response.^7^ In an animal model of MS, increased axonal injury was observed after systemic inflammatory challenge, and was associated with a damaging microglial phenotype.^7^ If systemic inflammation is confirmed to be a driver of progression, this would offer new avenues for intervention to slow disease progression. However, it remains to be shown that systemic inflammation is related to tissue loss in humans with MS.

To address this gap in knowledge, we investigated the association between systemic inflammation and CNS atrophy using magnetic resonance imaging (MRI) in a prospective longitudinal study. We hypothesized that systemic inflammation would correlate with loss of tissue volume. The MRI measures were selected due to their association with clinical outcome, and included atrophy of the spinal cord^8^ and brain.^9^ Systemic inflammation was quantified through measurement of urinary neopterin.^10^ Neopterin is produced after IFN-γ activation and is a non-specific marker of systemic inflammation; levels in the systemic compartment are independent of CNS inflammation.^11^ It can be conveniently measured in urine due to high renal clearance, and expression as a ratio to urinary creatinine controls for glomerular filtration rate and hydration status. These qualities make urinary neopterin an ideal biomarker for monitoring of systemic inflammation in longitudinal studies.^10^

The effects of a systemic inflammatory challenge on tissue injury depend on prior exposure to inflammation and may be markedly different depending on the duration and magnitude of the previous exposure, ranging from ‘priming’ to ‘tolerance’. This property of the innate immune system, termed ‘innate immune memory’, occurs as a result of transcriptional re-programming via epigenetic changes.^12^ For example, single^13^ or very low^14^ doses of lipopolysaccharide induce higher subsequent responses with tissue damage, while multiple^13^ or higher^14^ doses have the opposite (protective) effect. For these reasons, systemic inflammation was modelled as two separate variables: the inflammatory response (UNCR peaks) and background levels of systemic inflammation (background UNCR level).

## Methods

### Study design and participants

The SIMS (Systemic Inflammation in Multiple Sclerosis) study was a longitudinal cohort observational study conducted at the Wessex Neurological Centre, University Hospital Southampton NHS Foundation Trust, a tertiary neurological centre on the south coast of England (National Research Ethics Service approval 12SC0176 and institutional research ethics approval ERGO5562). Participants gave written informed consent. Recruitment occurred between January 2015 and September 2017. Inclusion criteria were: (i) diagnosis of primary or secondary progressive MS; (ii) age ≤ 70; (iii) EDSS score ≤ 6.5; and (iv) availability of a home freezer for storing urine samples. Exclusion criteria were: (i) relapse in the last year; (ii) disease-modifying or immunosuppressive treatment in the previous six months; (iii) comorbidities that could contribute to neurological disability; and (iv) pregnancy. *A priori*, it was planned that participants with on-study relapses would be excluded from the analysis, due to possible associations with systemic inflammation and progression, which would have complicated the analysis. In summary, this study was designed to focus exclusively on people with relapse-free progression. Primary progressive MS was defined according to the 2010 McDonald criteria.^15^ Secondary progressive MS was defined as sustained and steady progression in the preceding two years, confirmed by either an increase of at least one EDSS point or clinical documentation of increasing disability, where such worsening was not relapse-driven, similar to contemporary studies.^16,17^ A relapse was defined as patient-reported symptoms or objective signs typical of an acute inflammatory demyelination in the CNS, lasting 24 hours or more.^15,18^ The null hypothesis was that systemic inflammation, as measured by urinary neopterin, did not correlate with MRI measures of atrophy in cervical spinal cord and brain. Previous studies had shown that significant changes in cervical cord cross-sectional area and brain volume can be detected over two years with a sample size of 40 participants,^19,20^ so we aimed to recruit a similar number.

Participants were seen at six-monthly intervals for a total follow-up duration of 2.5 years. At the first timepoint, participants received verbal and written information about the study, written consent was obtained, baseline data were collected, and urine collection kits were provided. Participants collected weekly urine samples and stored them in their home freezer, returning them at subsequent visits.

### Clinical measures

Clinical disability was assessed six-monthly using the Multiple Sclerosis Functional Composite (MSFC),^21^ Expanded Disability Status Scale (EDSS),^22^ Multiple Sclerosis Impact Scale-29 (MSIS-29)^23^ and daily step count recorded over a 6-day period using a wearable device (SenseWear Armband, Model MF-SW, Body Media, Pittsburgh, PA).^24^ The MSFC *Z*-scores were normalized using the study baseline scores.^21^ All clinical measurements were performed by trained and certified research staff.

### MRI measures

MRI was performed at baseline and study exit, on a Siemens Skyra 3T MRI (Siemens Healthineers, Erlangen, Germany) using a 20-element phased-array head and neck coil, for a total scan time of 54 minutes. Due to the long duration of the study, scans at entrance and exit were naturally interleaved.

Structural images of the brain were acquired using a 3D magnetization-prepared rapid gradient echo (MP-RAGE) sequence with the following parameters: TR = 2200 ms, TE = 2.45 ms, TI = 900 ms, flip angle = 8°, GRAPPA undersampling with parallel imaging factor = 2, field of view of 250 × 250 × 176 mm^3^ and isotropic voxel resolution of 1.0 mm^3^. This sequence has excellent grey-white matter contrast, suitable for volume and atrophy estimation and tissue classification.^25^ All raw images were visually inspected by an experienced neuroradiologist.

Imaging processing was conducted in FSL.^26^ After cropping the field of view (using ‘robustfov’), brain extraction was performed with ‘BET’ with a fractional intensity threshold of 0.1 and applying bias field removal and standard-space masking, as recommended for MS.^27^ Images were lesion-filled prior to brain volume measurements to reduce segmentation errors due to T1 hypointense lesions.^28^ Annualized percentage change of brain volume between two timepoints was estimated with ‘SIENA’.^29^

Brain lesions were segmented using the lesion growth algorithm^30^ from the lesion segmentation toolbox (LST, version 20.0.15), operating within SPM (version 12) in MATLAB (Mathworks, Natick, MA, USA), on an axial fluid-attenuated inversion recovery (FLAIR) sequence covering the whole brain with the following parameters: TR = 5000 ms, TE = 397 ms, TI = 1800 ms, field-of-view = 256 × 248 × 194 mm^3^, voxel size 1.0 × 1.0 × 1.1 mm^3^, 176 slices.

The spinal cord was imaged using 3D MP-RAGE from the fourth ventricle to T4 with the following parameters: TR = 2200 ms, TE = 2.45 ms, TI = 900 ms, flip angle = 8°, GRAPPA undersampling with parallel imaging factor = 2, field of view 176 × 250 × 250 mm^3^, isotropic voxel resolution of 1.0 mm^3^. The mean spinal cord cross-sectional area between C2 and C5 levels was computed using ‘PropSeg’,^31^ part of the Spinal Cord Toolbox^32^; annualized percentage change between timepoints was calculated. This method for spinal cord volumetry is validated and has low scan-rescan variability.^8^ The analysis pipeline was developed in MATLAB running within Linux Red Hat 7 (Red Hat Inc, Raleigh, NC, USA).

### Urine collection and analysis

Urinary excretion of neopterin shows diurnal variation with maximum excretion in the early morning.^33^ Participants were trained to collect a first morning midstream urine sample on the same day once a week (using urine Monovettes from Sarsted, Nümbrecht, Germany), document the sampling date on a pre-prepared tube label, wrap the tube in aluminium foil (to shield the sample from light since neopterin is photosensitive) and store immediately in a −20°C home freezer. Participants received weekly text reminders to collect urine samples. Samples were brought to study appointments every three or six months. As part of quality control, these samples were checked and participants’ technique was assessed during clinic visits. Sample dates were monitored to ensure the weekly sampling schedule was being adhered to. Mean weekly urine collection rate was 88.1% (±10.1%). Upon sample receipt, urine was thawed and centrifuged at 10°C and 2000 g for 5 minutes. Aliquots were stored at −80°C until analysed.

Urinary neopterin and creatinine were measured using a validated ultra-performance liquid chromatography mass spectrometry technique, and the UNCR computed, as previously described.^10^ Briefly, urine samples were diluted in running buffer, 0.2% formic acid (primary mobile phase) and 0.2% formic acid in acetonitrile (co-solvent) (Fisher, UK) and kept in the dark at 5°C. Samples were analysed using a Waters ACQUITY UPLC interfaced with a Waters Xevo triple quadrupole mass spectrometer equipped with an electrospray ionization (ESI) probe, column oven and autosampler. Separation was achieved using a Waters UPLC penta-fluoro-phenyl (PFP, 1.7 µm, 2.1 × 100 mm) column, held at 24°C. After separation, the compounds (creatinine, neopterin and their internal standards, creatinine-d_3_ and neopterin ^13^C_5_) were monitored in scheduled multiple-reaction monitoring (MRM) mode with ESI-positive ionization, cone energy of 30 V and collision energy of 20 V. Concentration of analyte was calculated by integrating the area under the peaks using MassLynx v4.1 software and expressed as the urinary neopterin-to-creatinine ratio (UNCR) measured in µmol/mol.

### Quantifying inflammation over time

**Figure 1:**
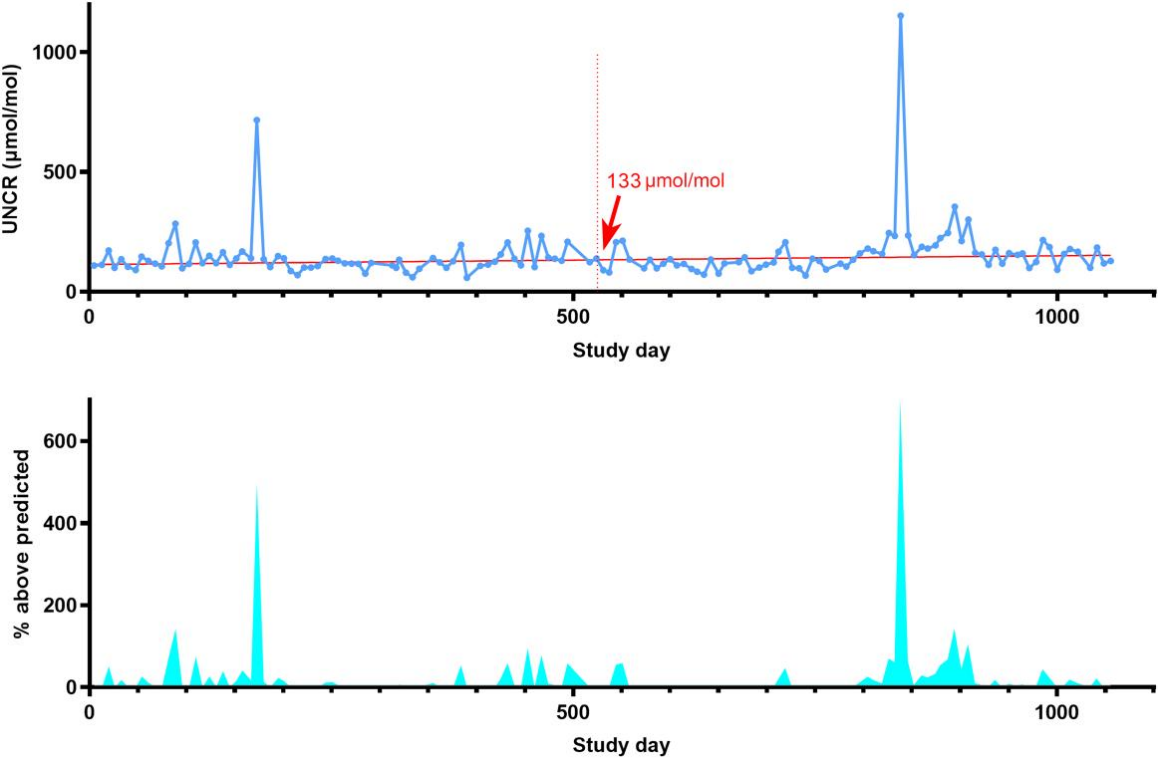
**Longitudinal UNCR analysis demonstrated from a single representative case.** The *top* panel shows the urinary neopterin-to-creatinine ratio (UNCR) time series (blue data points) and regression line (straight red line). The midpoint of the regression line (133 µmol/mol) is indicated (vertical dotted red line) and was used as a measure of background inflammation. There is a positive long-term gradient in the regression line. The obvious peaks (Days 173 and 838) were associated with symptomatic respiratory tract infections. The *bottom* panel shows the height of points above the regression line expressed as percentage above predicted value. The area under this curve (shaded, cyan) was used as a measure of the inflammatory response, incorporating the number, height and duration of peaks, separated from the inter-individual variation in background inflammation.

In preliminary work we noted significant inter-individual variation of UNCR time series with respect to two main components: (i) background UNCR—henceforth referred to as ‘background inflammation’; and (ii) short-term UNCR peaks, some of which were temporally associated with clinically apparent stimuli, such as symptomatic systemic infections, while others were asymptomatic^10^—henceforth referred to as the ‘inflammatory response’. Analysis of longitudinal UNCR data to quantify background inflammation and the inflammatory response was performed using a custom-built script in MATLAB. For each participant, the UNCR time series was plotted and a regression line fitted using the iteratively reweighted least squares method (to reduce the effect of peaks on the regression line). The midpoint of the regression line was reported as a measure of the participants’ background inflammation. Each UNCR value above the regression line was then expressed as a percentage height above the predicted value. The trapezoidal area-under-the-curve of peak height percentages was reported as a measure of the inflammatory response, and then annualized. A representative case is illustrated in Fig. 1.

### Statistical analysis

Statistical analyses were performed using SPSS version 27 (IBM Corp., Armonk, NY, USA). All measures of change were annualized to account for differences in follow-up duration. Data distribution was determined graphically and using Kolmogorov–Smirnov tests. Means and standard deviations are here given for parametric variables and medians and interquartile ranges for non-parametric data. All hypothesis testing was conducted at the two-tailed 5% significance level. To assess progression between baseline and follow-up, paired Student’s *t*-tests or paired Wilcoxon signed rank tests (for parametric and non-parametric data, respectively) were used. A one sample Student’s *t*-test was used to assess whether annualized MRI measures were significantly different from zero. Linear regression techniques were used to investigate the relationship between outcome measures and (i) background inflammation and (ii) inflammatory response. Since priming and/or preconditioning effects are known to occur in the CNS,^34-38^ the interaction between background inflammation and the inflammatory response was studied. In sensitivity analyses, the robustness of findings to the addition of covariates was tested. The selection of covariates was based on two considerations. First, the sample size limits the maximum number of variables, minimizing overfitting. Second, the aim of this study was to test a hypothesis (specifically to determine the effect of systemic inflammation on atrophy) rather than account for the maximum variance in outcome measures (as would be the case if one were to construct a model for prognostication). Hence, covariates were determined *a priori* using direct acyclic graph theory as those with robust evidence for a confounding effect, i.e. a documented causal path from these potential confounders to both exposure and outcome. These criteria were met by age and sex, which have been shown to affect both UNCR^10^ and neurodegeneration.^39^

## Results

### Study population characteristics

**Figure 2:**
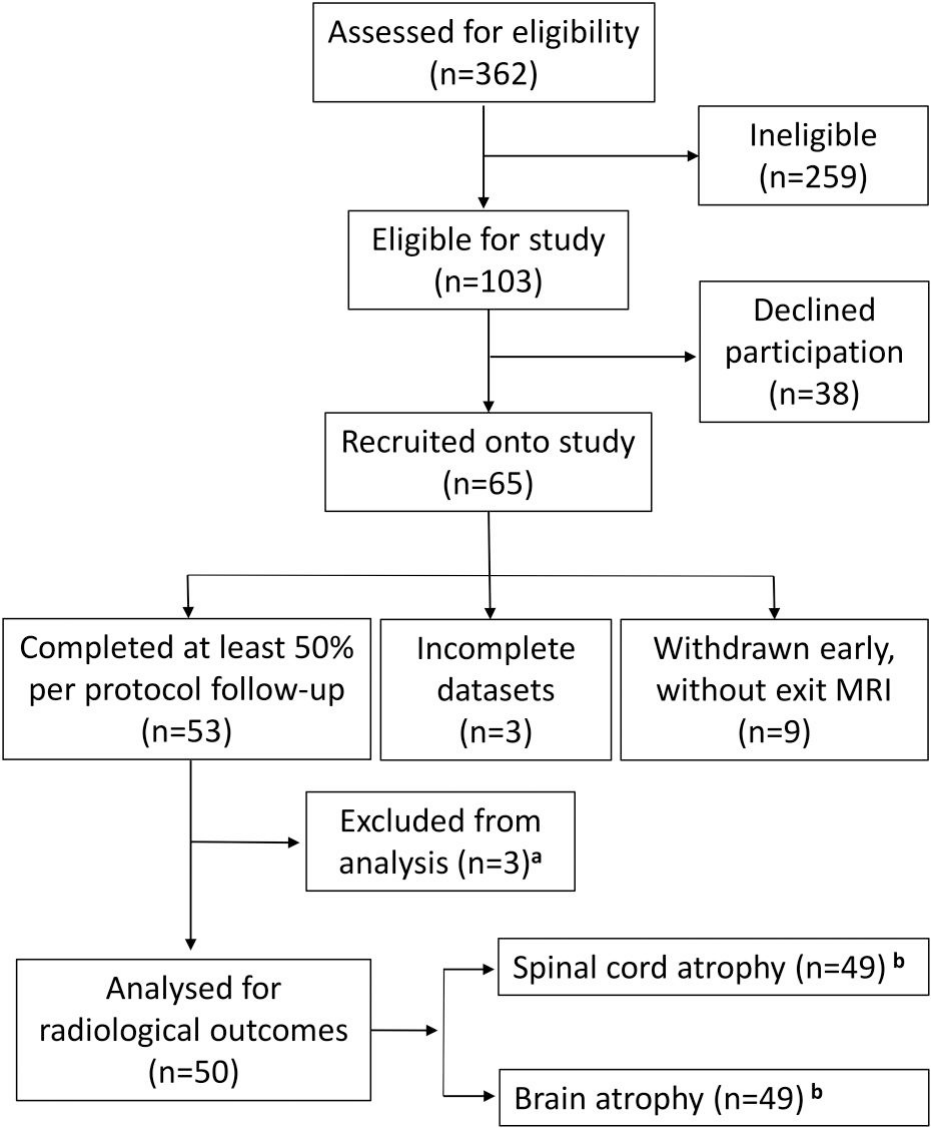
**SIMS (Systemic Inflammation in Multiple Sclerosis) study participant flow diagram.** The aim of this prospective longitudinal study was to investigate the association between systemic inflammation and (i) brain atrophy and (ii) spinal cord atrophy in non-relapsing progressive multiple sclerosis. Systemic inflammation was quantified as the urinary neopterin-to-creatinine ratio (UNCR). Atrophy was measured on magnetic resonance images (MRI) at the beginning and end of the study. ^a^One participant experienced a relapse accompanied by gadolinium enhancement on MRI, one participant had UNCR analyses that failed internal quality control due to spuriously low creatinine values and all MR images from one participant were unsuitable for analysis due to motion artefact. ^b^One participant’s MR images were unsuitable for brain atrophy measurement and one for cervical cord atrophy measurement.

**Figure 3:**
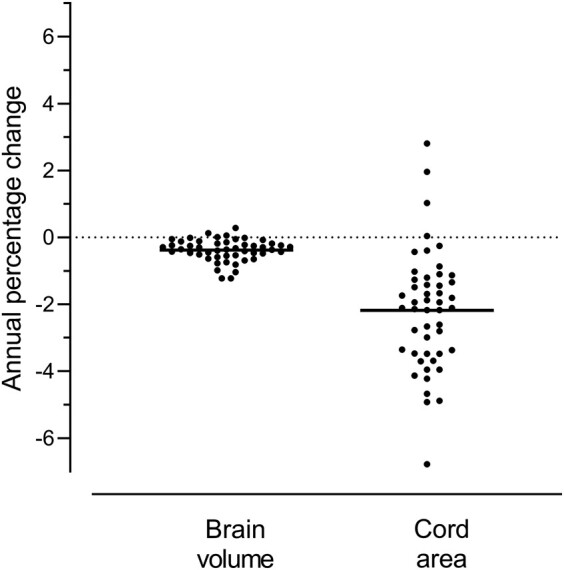
**Annualized percentage change in MRI measures.** A one sample *t*-test was used to assess progression (*n* = 49 individuals for brain atrophy and *n* = 49 individuals for cord atrophy). Both MRI measures showed significant progression (for brain atrophy: *t* = 8.170, df = 48, *P* < 0.001; for cord atrophy: *t* = 8.673, df = 48, *P* < 0.001), with cord atrophy exhibiting the highest mean annualized percentage change from baseline.

Fifty-three participants completed the study. Three cases were excluded due to a relapse on the study, gross motion artefact during imaging and spurious creatinine values. Hence, data from 50 participants were used in the analysis (Fig. 2). Mean follow-up duration was 2.6 years (range: 1.3–3.2 years). There were 28 (56%) individuals with PPMS and 22 (44%) with SPMS, 29 (58%) were female and the mean age was 53.4 (±8.0). This was a cohort with established disease (mean duration 12.4 years) and significant disability (74% had EDSS score ≥ 6.0, indicating the need for assisted ambulation). Other than the excluded case, none of the 50 participants developed relapses on the study, or received any disease-modifying treatment. T2 lesion count was not significantly different between the two timepoints (means of 19.8 and 20.2 lesions at the start and end, respectively, *P* = 0.473 in a paired *t*-test). Significant disability progression was observed clinically during the study period, as reflected by changes in the EDSS, MSFC and MSIS-29 (Table 1). Progression in this study population mainly affected limb function, compared to higher mental functions. Significant deterioration occurred in the timed 25-foot walk and nine-hole peg test, but not the Paced Auditory Serial Addition Test, when considering the MSFC (Table 1). Similarly, progression was observed in the physical, not psychological, sub-scores of the MSIS-29 (Table 1). Finally, a significant decrease in daily step count was observed (Table 1). The largest progression effect sizes were seen with MSFC, specifically the T25FW. Both brain and cervical cord atrophy occurred on the study, similar to rates published in previous studies,^19,41^ but the most striking change was seen in the cervical spinal cord (Fig. 3). This is in keeping with the corticospinal tract involvement that is typical of progressive disease.^42^ Since most of the progression in the study population clinically localized to the spinal cord, we next examined associations of systemic inflammation with cord atrophy.

**Table 1:** Baseline and mean annualized change in clinical scores

	Baseline		Mean annualized change over study			
Clinical variable	Mean (SD)	Median (IQR)	Mean annualized change (±SD)	95% confidence intervals for annualized change (Lower)–(Upper)	Test statistic (*t* or *Z*)^a^	Significance (effect size)^a^
EDSS score^b^	5.6 (1.4)	6.0 (0.5)	0.34 (0.66)	(0.15)–(0.53)	−3.34	**0.0004 (0.48)**
EDSS 0–5.5, No.	12					
EDSS 6.0–6.5, No.	37					
MSFC, *Z*-score	0.06 (0.7)	0.1 (1.2)	−0.63 (1.43)	(−1.04)–(−0.23)	−3.72	**0.0001 (0.53)**
T25FW, seconds	12.7 (8.7)	9.7 (7.0)	17.32 (42.5)	(5.24)–(29.4)	−3.64	**0.0002 (0.52)**
9HPT, seconds^c^	34.4 (52.9)	25.4 (9.2)	23.79 (116.78)	(−9.4)–(56.98)	−2.45	**0.014** (**0.35)**
PASAT, number correct	43.1 (12.9)	47.0 (22.5)	−1.1 (6.3)	(−2.89)–(0.69)	−0.95	0.35 **(**0.14)
MSIS-29 physical, points	47.9 (13.0)	49.0 (19.0)	2.58 (7.73)	(0.38)–(4.78)	2.36^+^	**0.022 (0.33)**
MSIS-29 psychological, points	17.9 (5.9)	17.0 (9.8)	1.34 (4.83)	(−0.03)–(2.71)	1.96^+^	0.060 (0.28)
Step count, steps/day^d^	3617.6 (3007.9)	2574.8 (3162.6)	−768.26 (1814.14)	(−1300.91)–(−235.61)	−3.08	**0.002 (0.45)**

SD, standard deviation; IQR, interquartile range; EDSS, Expanded Disability Status Scale; MSFC, Multiple Sclerosis Functional Composite; T25FW, timed 25-foot walk; 9HPT, nine-hole peg test; PASAT, Paced Auditory Serial Addition Test; MSIS-29, Multiple Sclerosis Impact Scale.

^a^Progression was assessed using paired tests (Student’s paired *t*-tests for parametric data, denoted by ‘+’, or paired Wilcoxon signed rank tests for non-parametric data) using end-point and baseline values and effect sizes calculated by Cohen’s *D* (parametric data) or by dividing the Wilcoxon *Z* by the square root of the number of observations (*Z*/√*n*, non-parametric data).^40^ Significant changes (*P*-values) are highlighted in bold.

^b^Baseline EDSS unavailable for one participant since accurate assessment was not possible due to a co-existing ankle problem.

^c^Average of both hands.

^d^Two participants were allergic to the physical activity monitor used to measure step count, one from baseline, one from six months.

### Systemic inflammation associates with cervical cord atrophy

**Figure 4:**
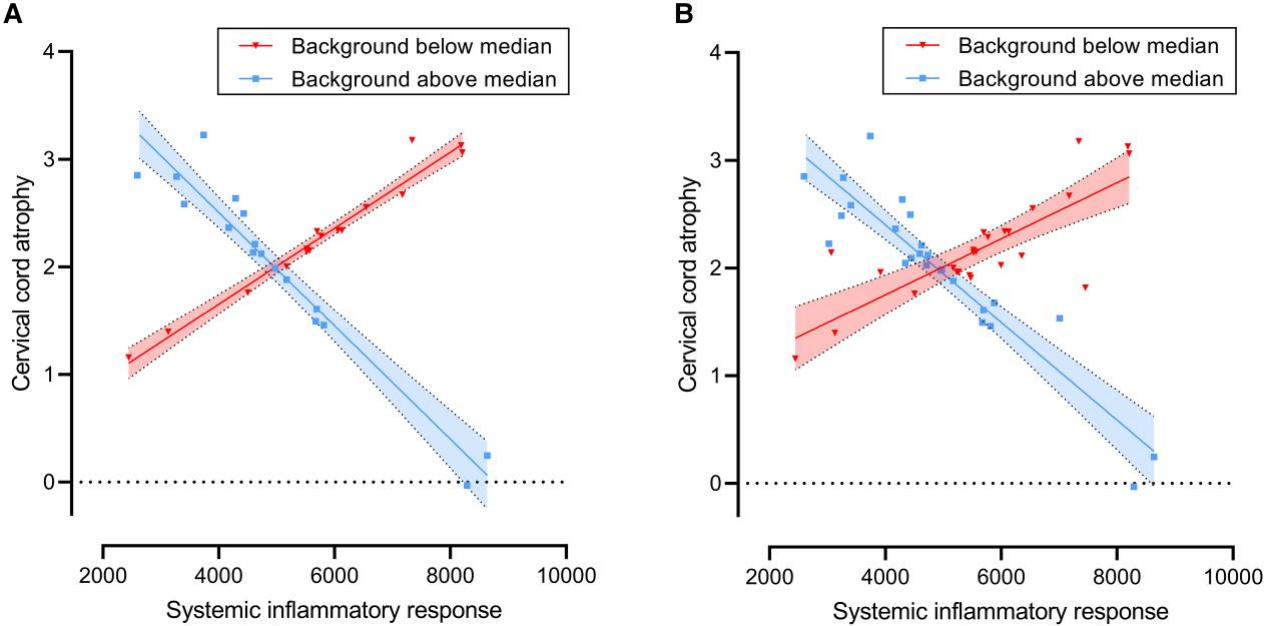
**Systemic inflammation is associated with cervical cord atrophy.** Systemic inflammation was modelled as a system of two variables: the inflammatory response and background inflammation. To visualize the interaction between the two inflammatory variables, individuals in the lower tertile (*n* = 16 individuals) and upper tertile (*n* = 17 individuals) of background inflammation are plotted separately (**A**). The whole dataset (*n* = 49 individuals), split into two equally-sized groups with background inflammation above (blue, square data points) and below (red, triangular data points) the median of the whole dataset, is shown in **B**. Predicted annualized cervical cord atrophy is shown on the *y*-axis and the inflammatory response is shown on the *x*-axis. The inflammatory response is quantified as annualized area under the curve of peak height percentages above baseline (see ‘Methods’). 95% confidence intervals are shown between dotted lines.

We independently modelled the contribution of background inflammation and the inflammatory response as well as their interaction, since priming and/or preconditioning phenomena have been documented in the CNS.^34-38^ In order to test the hypothesis that systemic inflammation was associated with cord atrophy, multivariable regression was performed with annualized percentage change in cervical cord cross-sectional area as outcome and the following systemic inflammatory variables: background inflammation, inflammatory response and their interaction. All variables of systemic inflammation significantly predicted cord atrophy (background inflammation: *P* = 0.03, standardized β = −1.23, inflammatory response: *P* = 0.03, standardized β = −1.34, interaction: *P* = 0.02, standardized β = 1.68, model *R*^2^ = 0.12, Table 2). While the degree of atrophy varied with the inflammatory response, this effect was modulated by background inflammation. A higher inflammatory response was associated with more pronounced cervical cord atrophy in the presence of background inflammation below the median (Fig. 4, red data points), but was associated with less marked cervical cord atrophy in the presence of background inflammation above the median (Fig. 4, blue data points). Variables of systemic inflammation did not significantly predict brain atrophy (background inflammation: *P* = 0.68, β = −0.26, inflammatory response: *P* = 0.62, β = −0.33, interaction: *P* = 0.80, β = 0.19, Table 2).

**Table 2:** Regression of MRI outcome measures on systemic inflammation

Dependent variable	Independent variable	Unstandardized coefficients		Standardized β coefficients	*t*-Statistic	Significance (two-tailed)	95.0% confidence interval for B		Effect size
		B	Standard error				Lower bound	Upper bound	
Annualized cervical cord atrophy	Constant	5.815	4.009		1.450	0.154	−2.260	13.890	
	Background UNCR	−4.525 × 10^−2^	2.062 × 10^−2^	−1.233	−2.194	**0**.**033**	−8.679 × 10^−2^	−3.72 × 10^−3^	0.10
	UNCR response	−1.55 × 10^−3^	7.03 × 10^−4^	−1.341	−2.199	**0**.**033**	−2.96 × 10^−3^	−1.30 × 10^−4^	0.10
	Interaction	8.91 × 10^−6^	3.73 × 10^−6^	1.684	2.392	**0**.**021**	1.41 × 10^−6^	1.64 × 10^−5^	0.13
	Overall model: *F*(3,48) = 2.03, *P* = 0.12, *R*^2^ = 0.12								
Annualized brain atrophy	Constant	0.145	0.834		0.174	0.863	−1.535	1.825	
	Background UNCR	−1.77 × 10^−3^	4.22 × 10^−3^	−0.26	−0.419	0.677	−1.03 × 10^−2^	6.73 × 10^−3^	0.00
	UNCR response	−7.18 × 10^−5^	1.44 × 10^−4^	−0.329	−0.498	0.621	−3.62 × 10^−4^	2.18 × 10^−4^	0.01
	Interaction	1.91 × 10^−7^	7.52 × 10^−7^	0.19	0.253	0.801	−1.33 × 10^−6^	1.71 × 10^−6^	0.00
	Overall model: *F*(3,48) = 0.46, *P* = 0.71, *R*^2^ = 0.03								

Annualized cervical cord atrophy and brain atrophy were dependent variables. Background urinary neopterin-to-creatinine ratio (UNCR), UNCR response and their interaction were predictor variables. *P* values < 0.05 are shown in bold. Effect size was computed using Cohen’s *f*^2^.^43^

### Sensitivity analyses

Controlling for the potential confounders of age and sex showed that the effect of systemic inflammation on MRI measures was robust (Table 3). The mean rate of lesion accrual was slow (0.17 ± 1.89 lesions per year, mean ± SD). However, 15 (30%) subjects accrued on average at least 1 lesion per year. In a sensitivity analysis excluding these subjects, the model for prediction of spinal cord atrophy performed better than when using the whole cohort, both in the unadjusted model (background inflammation: *P* = 0.02, standardized β = −1.40, inflammatory response: *P* = 0.02, standardized β = −1.82, interaction: *P* = 0.01, standardized β = 2.25, model *R*^2^ = 0.46) and the model including age and sex as confounders (background inflammation: *P* = 0.01, standardized β = −1.59, inflammatory response: *P* = 0.01, standardized β = −1.87, interaction: *P* = 0.006, standardized β = 2.41, model *R*^2^ = 0.29).

**Table 3:** Sensitivity analysis

Dependent variable	Independent variable	Unstandardized coefficients		Standardized β coefficients	*t*-Statistic	Significance (two-tailed)	95.0% confidence interval for B		Effect size
		B	Standard error				Lower bound	Upper bound	
Annualized cervical cord atrophy	Constant	6.07	4.22		1.437	0.158	−2.45	14.58	
	Background UNCR	−4.57 × 10^−2^	2.23 × 10^−2^	−1.246	−2.048	**0**.**047**	−9.07 × 10^−2^	−7.01 × 10^−4^	0.094
	UNCR response	−1.54 × 10^−3^	7.27 × 10^−4^	−1.336	−2.117	**0**.**040**	−3.00 × 10^−3^	−7.31 × 10^−5^	0.101
	Interaction	8.90 × 10^−6^	3.91 × 10^−6^	1.681	2.279	**0**.**028**	1.03 × 10^−6^	1.68 × 10^−5^	0.119
	Age	−5.31 × 10^−3^	3.44 × 10^−2^	−0.025	−0.155	0.878	−7.47 × 10^−2^	6.40 × 10^−2^	0.000
	Sex	0.17	0.60	0.048	0.285	0.777	−1.04	1.38	0.002
	Overall model: *F*(5,48) = 1.2, *P* = 0.33, *R*^2^ = 0.12								

Regression results for annualized cervical cord atrophy as independent variable, and background urinary neopterin-to-creatinine ratio (UNCR), UNCR response and their interaction as predictor variables, with age and sex as confounding covariates. *P* values < 0.05 are shown in bold. Effect size was computed using Cohen’s *f*^2^, as in Table 2.

### Exploratory analyses

Although the study was not adequately powered for a clinical primary outcome, an exploratory analysis of the correlation between systemic inflammation and clinical measures (MSFC, EDSS, MSIS-29 and step count) was still conducted since the data were available, but no association was detected (data not shown).

### Time course

**Figure 5:**
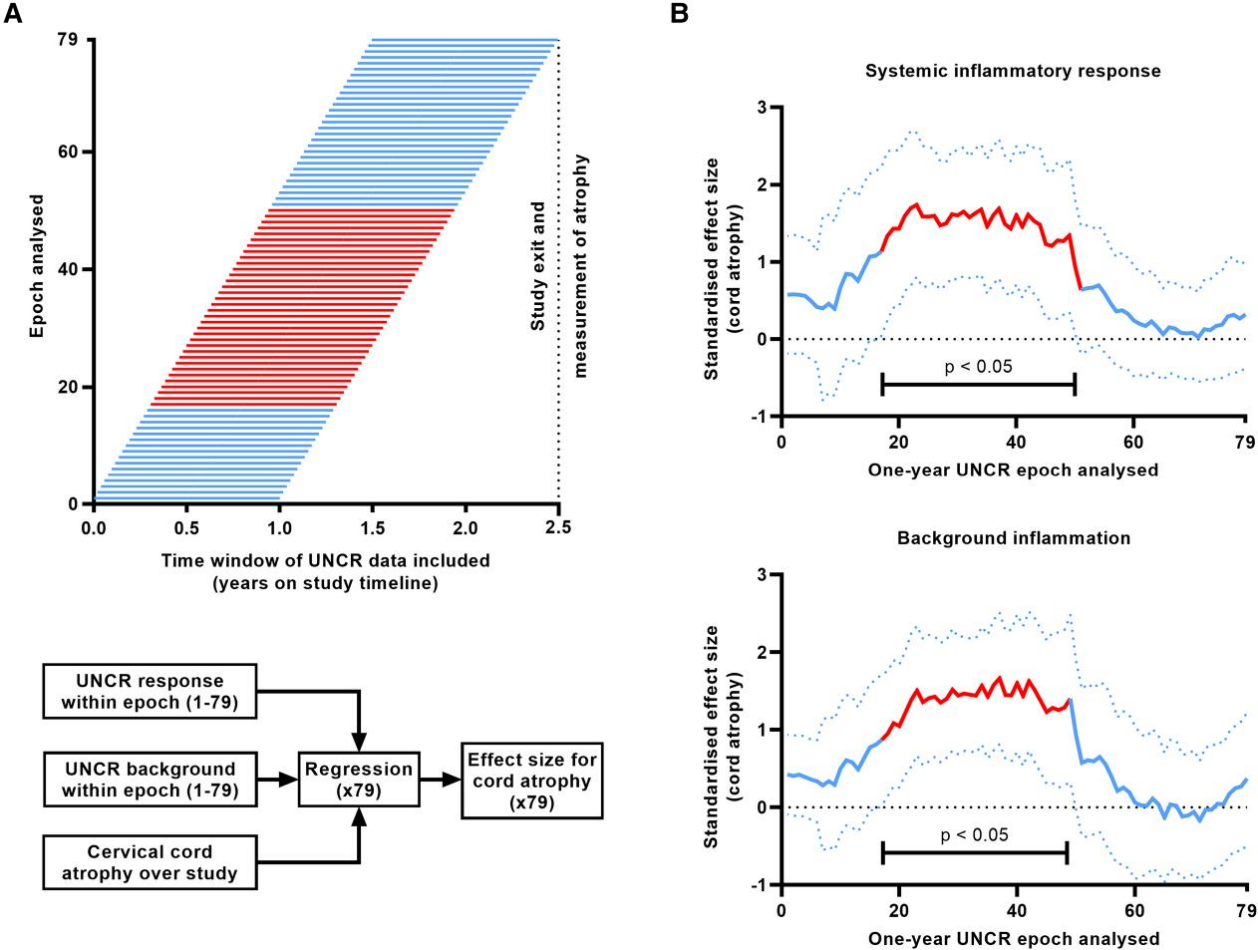
**Delay between systemic inflammation and subsequent cervical cord atrophy. A** outlines the method for analysis of urinary neopterin-to-creatinine ratio (UNCR) time series data in one-year epochs. Each horizontal line represents one of 79 one-year UNCR epochs, frame-shifted one week apart. For each UNCR epoch, inflammatory variables within this epoch from all participants were passed into a multivariable regression with cord atrophy as the outcome variable. The epochs coloured red (epochs 17-50) showed a significant effect (*P* < 0.05) as **B** elaborates further. **B** shows the effect size and 95% confidence intervals for the systemic inflammatory response (upper plot) and background inflammation (lower plot) on cervical cord atrophy, according to one-year UNCR epochs. The plots represent line graphs of connected individual data points (*n* = 79 UNCR epochs) where each data point is the effect size of a single one-year UNCR epoch. In both cases, systemic inflammation in the middle third of epochs was significantly associated with cord atrophy (epochs 17–50 ending 29 weeks prior to study exit for the systemic inflammatory response and epochs 17–49 ending 30 weeks prior to study exit for background inflammation). This association was lost for: (i) the early epochs, probably because their effect was overshadowed by later epochs; and (ii) the last third of epochs, suggesting that a delay is essential for the association between systemic inflammation and cord atrophy.

Since follow-up was for 2.5 years, further analysis was conducted to determine the length of time needed for systemic inflammation to affect cord atrophy. For each individual, annualized values for background inflammation and the inflammatory response were re-calculated after restricting the UNCR time series to a one-year epoch. The first epoch was the one-year window beginning 2.5 years prior to study exit, while the last epoch was the one-year window ending on the day of study exit. Intervening one-year epochs were created by incrementally shifting the time window by one week; in total, one could analyse 79 one-year epochs covering the 2.5 year study duration (Fig. 5A). If an individual did not have data in the specified epoch due to limited follow-up duration, they were excluded from that iteration; this applied to four individuals (8%). For each epoch, a multivariable regression was conducted with the inflammatory variables as predictors and cord atrophy as the outcome. The effect size plotted across the rolling epochs is shown in Fig. 5B. The association between systemic inflammation and cord atrophy is clearly shown but was not significant for the last third of epochs (Fig. 5B). This was the case for both the inflammatory response and background inflammation (Fig. 5B). This is in keeping with a time lag between systemic inflammation and cord atrophy.

## Discussion

In this longitudinal study following individuals with established progressive MS over a 2.5-year study period, we observed a robust association between markers of increased systemic inflammation and cord atrophy. Association does not equate to causation and reverse causation needs to be considered, for example increasing cord atrophy may lead to higher levels of systemic inflammation because of a greater risk of infections with more disability. However, temporal analysis showed that the inflammatory response was only associated with cord atrophy when it pre-dated study exit imaging by ∼30 weeks (Fig. 5B). Axon/myelin loss is a slow process, so such a delay would be expected if systemic inflammation was causative, due to the need for the tissue degeneration underlying atrophy to progress to a measurable extent. This time lag between the inflammatory response and cord atrophy argues against the association being a reverse causation.

Interaction plotting showed that a higher inflammatory response was associated with accelerated cord atrophy in the presence of background systemic inflammation below the median for the study population (Fig. 4). This is an important observation since it confirms that conditioning in humans is also sensitive to the dose of pre-existing inflammatory stimulus, with lower conditioning doses more likely to induce ‘priming’ as has been observed in experimental studies.^13,14^ Conversely, the data suggest that higher background inflammation, while leading to cervical cord atrophy itself, may subdue the tissue-damaging effects of subsequent acute inflammatory challenges. Such a ‘tolerizing’ preconditioning effect is well-recognized in the context of cerebral ischaemic^37^ and spinal cord injury, where prior exposure to inflammation promoted microglial activation to a reparative phenotype with subsequent improved functional outcomes.^34^ It is important to note that cervical cord atrophy occurred in all patients except for one (Fig. 4), so the interaction between background inflammation and the inflammatory response was associated with the extent of cord atrophy observed, rather than the occurrence of cord atrophy (Fig. 4).

Background inflammation in MS participants was not significantly different from non-MS controls in a published cohort,^10^ in an analysis of covariance adjusting for age and sex. The estimated marginal means for background UNCR was 216.7 (95% confidence interval: 201.0–232.4) for controls and 195.8 (95% confidence interval: 177.8–213.8) for the MS participants in this cohort (*P* = 0.093). It is therefore unlikely that MS disease activity contributed to UNCR. Indeed, a previous study demonstrated compartmentalization of the neopterin response, such that when cerebrospinal fluid neopterin increased in MS, it was not reflected in the serum.^11^ Moreover, in our study, there was no association between T2 lesion volume change and systemic inflammation as measured with UNCR, whether background inflammation or inflammatory response (data not shown).

In this study, the aetiology of the inflammatory response (UNCR peaks) was not determined. In a previous study,^10^ we followed the time course of UNCR in young and elderly healthy controls, while recording symptoms and possible sources of sterile inflammation such as injuries or surgery. Some peaks were associated with clinically apparent infections, while many peaks were asymptomatic. The aetiology of these asymptomatic peaks remains to be determined; possibilities include stochastic perturbations in the immune response, asymptomatic viraemia or bacteraemia, or sterile inflammatory stimuli.^10^

An important question arises: why did systemic inflammation associate with spinal cord and not brain atrophy in our study? Several reasons may underlie this observation. In our population of progressive MS, most active progression occurred in the limbs, with little change in cognition for example, suggesting that spinal pathology was the dominant driver of progression. A higher level of microglial activation in the spinal cord, compared to the brain, may have resulted in a greater susceptibility to systemic inflammation in the cord in the study population. Macroscopic and microscopic structural differences between the spinal cord and brain may explain a true biological differential susceptibility to systemic inflammation. For example, the spinal cord has a smaller cross-sectional area and inflammation spreading from the pial circulation is likely to affect a larger fraction of the cord parenchyma, compared to brain. The blood–spinal cord barrier is leakier than the blood–brain barrier,^44^ and these barriers play an important role in mediating the effects of systemic inflammation on the CNS.^45^ Finally, it is possible that brain atrophy was masked by an increase in brain volume associated with systemic inflammation, such that the relationship between systemic inflammation and brain atrophy was hard to detect. Inflammation may increase brain volume due to: (i) increased water content^46^ associated with widespread low level blood–brain barrier hyperpermeability especially in progressive MS;^47^ and (ii) increased microglial, astroglial or infiltrating leucocytes numbers.^48^ A similar phenomenon underlies the paradoxical increase in brain atrophy during first few months after starting anti-inflammatory disease-modifying agents, a phenomenon called ‘pseudo-atrophy’.^49^ While recent imaging techniques such as restriction spectrum imaging may be used to measure free water content,^50^ changes in glial and leucocyte components would be impossible to distinguish from neuroaxonal damage with MRI. A biochemical marker of neurodegeneration such as neurofilament-light^51^ may be a better readout of brain tissue damage than brain volume, and should be considered in future studies. The possibility that the spinal cord is especially susceptible to systemic inflammation is most intriguing and worthy of further investigation, not only from a scientific view but more importantly since cord atrophy has profound clinical consequences.

Both relapses and progression contribute to long-term disability.^52,53^ Several studies have demonstrated that systemic infections are associated with subsequent relapses^54-56^ and more frequently lead to disability compared with non-infection associated exacerbations.^55^ This study adds to the literature by demonstrating that systemic inflammation contributes to long-term disability progression in the absence of relapses, i.e. progression independent of relapse activity.

This study has a number of strengths and limitations. Findings were robust to sensitivity analyses. Weekly urine sampling was sustained with a high rate of compliance for the study’s duration, which shows that long-term inflammation monitoring using UNCR is practical and acceptable. More frequent sampling would have minimized the risk of failing to capture the true peak of the inflammatory response, particularly if this was short-lived. However, the study was designed with substantial patient and public input, and it was felt that more frequent sampling would have affected the participants’ experience and retention on the study. Neopterin was used to quantify systemic inflammation since the IFN-γ pathway is a master checkpoint for many cytokines in the inflammatory response.^57^ However, neopterin is more representative of a Th1-type immune response and possibly more responsive to cellular infections such as viral infections, compared to bacterial infections.^58^ Other markers of systemic inflammation would increase the robustness of future studies. The reliance on imaging at only two timepoints, the start and end of the study, was a limitation, as intermediate imaging timepoints improve power,^59^ minimize the effect of drop-out, enable recognition of non-linear trajectories of progression and allow a closer examination of the temporal relationship between progression and systemic inflammation. However, patient input during study design suggested that more MRI acquisitions would not be favoured by participants and could jeopardize their retention in this study that had a long duration.

In conclusion, this study has demonstrated that systemic inflammation is associated with tissue loss in MS along critical spinal cord pathways. However, causation is not proven, and further work is needed since efforts to minimize systemic inflammation in people with MS may be warranted, using a combination of vaccination, optimized symptom management and early treatment of infections. Further experimental interrogation of the hypothesis that systemic inflammation drives MS-related CNS atrophy is needed, for instance using preclinical models of progressive MS and experimental suppression and enhancement of systemic inflammation. Further clinical investigation is needed to study this phenomenon. Systemic infections are a common source of systemic inflammation, and commoner in MS.^60^ It would be challenging to undertake a comprehensive identification of all systemic infections in a longitudinal study with the purpose of relating these infections to long-term markers of progression such as brain or cord atrophy, given the lag in time between inflammation events and neurodegeneration. More pragmatically, future studies could focus on earlier effects on the brain, such as blood–brain barrier permeability,^61^ perfusion changes, or slowly expanding lesions,^62,63^ and their association with the systemic inflammatory signature of common infections such as those affecting the bladder. Lastly, immunosuppressive disease-modifying treatments target the adaptive immune response, more so than the innate immune response, yet the latter mediates the brain’s response to systemic inflammation.^12^ This may explain the lack of efficacy of immunosuppressive disease-modifying treatments on the progressive phase of MS. However, the identification of systemic inflammation as a driver of tissue damage in progressive MS offers a therapeutic opportunity, so further study of the underlying biology is needed.

## Data Availability

Data are available on request subject to ethical and institutional approvals.

## References

[1] James Lind Alliance Priority Setting Partnership . Multiple Sclerosis Top 10 Priorities from the James Lind Alliance Priority Setting Partnership in Multiple Sclerosis. https://www.jla.nihr.ac.uk/priority-setting-partnerships/multiple-sclerosis/top-10-priorities/. Accessed 1 May 2024.

[2] Trapp BD, Peterson J, Ransohoff RM, Rudick R, Mörk S, Bö L. Axonal transection in the lesions of multiple sclerosis. New Engl J Med. 1998;338(5):278–285. 9445407 10.1056/NEJM199801293380502

[3] Ferguson B, Matyszak MK, Esiri MM, Perry VH. Axonal damage in acute multiple sclerosis lesions. Brain. 1997;120(3):393–399. 9126051 10.1093/brain/120.3.393

[4] Davie CA, Barker GJ, Webb S, et al Persistent functional deficit in multiple sclerosis and autosomal dominant cerebellar ataxia is associated with axon loss. Brain. 1995;118(Pt 6):1583–1592. 8595487 10.1093/brain/118.6.1583

[5] Tallantyre EC, Bø L, Al-Rawashdeh O, et al Clinico-pathological evidence that axonal loss underlies disability in progressive multiple sclerosis. Mult Scler J. 2010;16(4):406–411. 10.1177/1352458510364992 20215480

[6] Strik M, Cofré Lizama LE, Shanahan CJ, et al Axonal loss in major sensorimotor tracts is associated with impaired motor performance in minimally disabled multiple sclerosis patients. Brain Communications. 2021; 3(2):fcab032. 34222866 10.1093/braincomms/fcab032 PMC8244644

[7] Moreno B, Jukes J-P, Vergara-Irigaray N, et al Systemic inflammation induces axon injury during brain inflammation. Ann Neurol. 2011;70(6):932–942. 22190366 10.1002/ana.22550

[8] Yiannakas MC, Mustafa AM, De Leener B, et al Fully automated segmentation of the cervical cord from T1-weighted MRI using PropSeg: Application to multiple sclerosis. Neuroimage Clin. 2016;10:71–77. 26793433 10.1016/j.nicl.2015.11.001 PMC4678307

[9] Miller DH, Barkhof F, Frank JA, Parker GJ, Thompson AJ. Measurement of atrophy in multiple sclerosis: Pathological basis, methodological aspects and clinical relevance. Brain. 2002;125(Pt 8):1676–1695. 12135961 10.1093/brain/awf177

[10] Stuart CM, Zotova E, Koster G, et al High-throughput urinary neopterin-to-creatinine ratio monitoring of systemic inflammation. J Appl Lab Med. 2019;5:101–113. 10.1373/jalm.2019.030007 31704895

[11] Fredrikson S, Link H, Eneroth P. CSF neopterin as marker of disease activity in multiple sclerosis. Acta Neurol Scand. 1987;75(5):352–355. 3618112 10.1111/j.1600-0404.1987.tb05458.x

[12] Neher JJ, Cunningham C. Priming microglia for innate immune memory in the brain. Trends Immunol. 2019;40(4):358–374. 30833177 10.1016/j.it.2019.02.001

[13] Wendeln A-C, Degenhardt K, Kaurani L, et al Innate immune memory in the brain shapes neurological disease hallmarks. Nature. 2018;556(7701):332–338. 29643512 10.1038/s41586-018-0023-4 PMC6038912

[14] Chen K, Geng S, Yuan R, Diao N, Upchurch Z, Li L. Super-low dose endotoxin pre-conditioning exacerbates sepsis mortality. eBioMedicine. 2015;2(4):324–333. 26029736 10.1016/j.ebiom.2015.03.001 PMC4445878

[15] Polman CH, Reingold SC, Banwell B, et al Diagnostic criteria for multiple sclerosis: 2010 revisions to the McDonald criteria. Ann Neurol. 2011;69(2):292–302. 21387374 10.1002/ana.22366 PMC3084507

[16] Chataway J, Schuerer N, Alsanousi A, et al Effect of high-dose simvastatin on brain atrophy and disability in secondary progressive multiple sclerosis (MS-STAT): A randomised, placebo-controlled, phase 2 trial. Lancet. 2014;383(9936):2213–2221. 24655729 10.1016/S0140-6736(13)62242-4

[17] Kapoor R, Furby J, Hayton T, et al Lamotrigine for neuroprotection in secondary progressive multiple sclerosis: A randomised, double-blind, placebo-controlled, parallel-group trial. Lancet Neurol. 2010;9(7):681–688. 20621711 10.1016/S1474-4422(10)70131-9

[18] Galea I, Ward-Abel N, Heesen C. Relapse in multiple sclerosis. BMJ: Br Med J. 2015;350:h1765. 25872511 10.1136/bmj.h1765

[19] Agosta F, Absinta M, Sormani MP, et al In vivo assessment of cervical cord damage in MS patients: A longitudinal diffusion tensor MRI study. Brain. 2007;130(8):2211–2219. 17535835 10.1093/brain/awm110

[20] Ingle GT, Stevenson VL, Miller DH, Thompson AJ. Primary progressive multiple sclerosis: A 5-year clinical and MR study. Brain. 2003;126(11):2528–2536. 12902314 10.1093/brain/awg261

[21] Fischer JS, Rudick RA, Cutter GR, Reingold SC. The Multiple Sclerosis Functional Composite Measure (MSFC): An integrated approach to MS clinical outcome assessment. National MS Society Clinical Outcomes Assessment Task Force. Mult Scler. 1999;5(4):244–250. 10467383 10.1177/135245859900500409

[22] Kurtzke JF . Rating neurologic impairment in multiple sclerosis: An expanded disability status scale (EDSS). Neurology. 1983;33(11):1444–1452. 6685237 10.1212/wnl.33.11.1444

[23] Hobart J, Lamping D, Fitzpatrick R, Riazi A, Thompson A. The Multiple Sclerosis Impact Scale (MSIS-29): A new patient-based outcome measure. Brain. 2001;124(Pt 5):962–973. 11335698 10.1093/brain/124.5.962

[24] Stuart CM, Varatharaj A, Domjan J, Philip S, Galea I. Physical activity monitoring to assess disability progression in multiple sclerosis. Mult Scler J Exp Transl Clin. 2020;6(4):2055217320975185. 10.1177/2055217320975185 PMC7727071 33343919

[25] Vrenken H, Jenkinson M, Horsfield MA, et al Recommendations to improve imaging and analysis of brain lesion load and atrophy in longitudinal studies of multiple sclerosis. J Neurol. 2013;260(10):2458–2471. 23263472 10.1007/s00415-012-6762-5 PMC3824277

[26] Smith SM, Jenkinson M, Woolrich MW, et al Advances in functional and structural MR image analysis and implementation as FSL. Neuroimage. 2004;23(Suppl 1):S208–S219. 15501092 10.1016/j.neuroimage.2004.07.051

[27] Popescu V, Battaglini M, Hoogstrate WS, et al Optimizing parameter choice for FSL-Brain Extraction Tool (BET) on 3D T1 images in multiple sclerosis. NeuroImage. 2012;61(4):1484–1494. 22484407 10.1016/j.neuroimage.2012.03.074

[28] Valverde S, Oliver A, Roura E, et al Quantifying brain tissue volume in multiple sclerosis with automated lesion segmentation and filling. NeuroImage: Clin. 2015;9:640–647. 26740917 10.1016/j.nicl.2015.10.012 PMC4644250

[29] Smith SM, Zhang Y, Jenkinson M, et al Accurate, robust, and automated longitudinal and cross-sectional brain change analysis. Neuroimage. 2002;17(1):479–489. 12482100 10.1006/nimg.2002.1040

[30] Schmidt P, Gaser C, Arsic M, et al An automated tool for detection of FLAIR-hyperintense white-matter lesions in Multiple Sclerosis. Neuroimage. 2012;59(4):3774–3783. 22119648 10.1016/j.neuroimage.2011.11.032

[31] De Leener B, Kadoury S, Cohen-Adad J. Robust, accurate and fast automatic segmentation of the spinal cord. Neuroimage. 2014;98:528–536. 24780696 10.1016/j.neuroimage.2014.04.051

[32] De Leener B, Lévy S, Dupont SM, et al SCT: Spinal Cord Toolbox, an open-source software for processing spinal cord MRI data. Neuroimage. 2017;145(Pt A):24–43. 27720818 10.1016/j.neuroimage.2016.10.009

[33] Auzeby A, Bogdan A, Krosi Z, Touitou Y. Time-dependence of urinary neopterin, a marker of cellular immune activity. Clin Chem. 1988;34(9):1866–1867. 3416434

[34] Hayakawa K, Okazaki R, Morioka K, Nakamura K, Tanaka S, Ogata T. Lipopolysaccharide preconditioning facilitates M2 activation of resident microglia after spinal cord injury. J Neurosci Res. 2014;92(12):1647–1658. 25044014 10.1002/jnr.23448

[35] Rosenzweig HL, Lessov NS, Henshall DC, Minami M, Simon RP, Stenzel-Poore MP. Endotoxin preconditioning prevents cellular inflammatory response during ischemic neuroprotection in mice. Stroke. 2004;35(11):2576–2581. 15375302 10.1161/01.STR.0000143450.04438.ae

[36] Rosenzweig HL, Minami M, Lessov NS, et al Endotoxin preconditioning protects against the cytotoxic effects of TNFalpha after stroke: A novel role for TNFalpha in LPS-ischemic tolerance. J Cereb Blood Flow Metab. 2007;27(10):1663–1674. 17327883 10.1038/sj.jcbfm.9600464

[37] Gidday JM . Cerebral preconditioning and ischaemic tolerance. Nat Rev Neurosci. 2006;7(6):437–448. 16715053 10.1038/nrn1927

[38] Biswas SK, Lopez-Collazo E. Endotoxin tolerance: New mechanisms, molecules and clinical significance. Trends Immunol. 2009;30(10):475–487. 19781994 10.1016/j.it.2009.07.009

[39] Guo JY, Isohanni M, Miettunen J, et al Brain structural changes in women and men during midlife. Neurosci Lett. 2016;615:107–112. 26777626 10.1016/j.neulet.2016.01.007 PMC4762229

[40] Rosenthal R . Parametric measures of effect size. The handbook of research synthesis. Russell Sage Foundation; 1994:231–244.

[41] Stefano D, Stromillo ML, Giorgio A, et al Establishing pathological cut-offs of brain atrophy rates in multiple sclerosis. J Neurol Neurosurg Psychiatry. 2016;87(1):93. 25904813 10.1136/jnnp-2014-309903 PMC4717444

[42] Kremenchutzky M, Rice GP, Baskerville J, Wingerchuk DM, Ebers GC. The natural history of multiple sclerosis: A geographically based study 9: Observations on the progressive phase of the disease. Brain. 2006;129(Pt 3):584–594. 16401620 10.1093/brain/awh721

[43] Cohen J . Statistical power analysis for the behavioral sciences. 2nd edn. LAWRENCE ERLBAUM ASSOCIATES; 1988.

[44] Bartanusz V, Jezova D, Alajajian B, Digicaylioglu M. The blood–spinal cord barrier: Morphology and clinical implications. Ann Neurol. 2011;70(2):194–206. 21674586 10.1002/ana.22421

[45] Galea I . The blood–brain barrier in systemic infection and inflammation. Cell Mol Immunol. 2021;18(11):2489–2501. 34594000 10.1038/s41423-021-00757-x PMC8481764

[46] Vavasour IM, Sun P, Graf C, et al Characterization of multiple sclerosis neuroinflammation and neurodegeneration with relaxation and diffusion basis spectrum imaging. Mult Scler. 2022;28(3):418–428. 34132126 10.1177/13524585211023345 PMC9665421

[47] Hochmeister S, Grundtner R, Bauer J, et al Dysferlin is a new marker for leaky brain blood vessels in multiple sclerosis. J Neuropathol Exp Neurol. 2006;65(9):855–865. 16957579 10.1097/01.jnen.0000235119.52311.16

[48] Lassmann H . Multiple sclerosis pathology. Cold Spring Harb Perspect Med. 2018;8(3):a028936. 29358320 10.1101/cshperspect.a028936 PMC5830904

[49] De Stefano N, Arnold DL. Towards a better understanding of pseudoatrophy in the brain of multiple sclerosis patients. Mult Scler J. 2015;21(6):675–676. 10.1177/1352458514564494 25623248

[50] Sowa P, Harbo HF, White NS, et al Restriction spectrum imaging of white matter and its relation to neurological disability in multiple sclerosis. Mult Scler J. 2019;25(5):687–698. 10.1177/1352458518765671 29542336

[51] Petzold A, Mondria T, Kuhle J, et al Evidence for acute neurotoxicity after chemotherapy. Ann Neurol. 2010;68(6):806–815. 21194151 10.1002/ana.22169

[52] Scalfari A, Neuhaus A, Daumer M, Muraro PA, Ebers GC. Onset of secondary progressive phase and long-term evolution of multiple sclerosis. J Neurol Neurosurg Psychiatry. 2014;85(1):67–75. 23486991 10.1136/jnnp-2012-304333

[53] Eriksson M, Andersen O, Runmarker B. Long-term follow up of patients with clinically isolated syndromes, relapsing-remitting and secondary progressive multiple sclerosis. Mult Scler J. 2003;9(3):260–274. 10.1191/1352458503ms914oa 12814173

[54] Sibley WA, Bamford CR, Clark K. Clinical viral infections and multiple sclerosis. Lancet. 1985;1(8441):1313–1315. 2860501 10.1016/S0140-6736(85)92801-6 PMC7173199

[55] Buljevac D, Flach HZ, Hop WC, et al Prospective study on the relationship between infections and multiple sclerosis exacerbations. Brain. 2002;125(Pt 5):952–960. 11960885 10.1093/brain/awf098

[56] Panitch HS . Influence of infection on exacerbations of multiple sclerosis. Ann Neurol. 1994;36(S1):S25–S28. 8017885 10.1002/ana.410360709 PMC7159629

[57] Zha Z, Bucher F, Nejatfard A, et al Interferon-γ is a master checkpoint regulator of cytokine-induced differentiation. Proc Natl Acad Sci U S A. 2017;114(33):E6867–E6874. 28760993 10.1073/pnas.1706915114 PMC5565454

[58] Denz H, Fuchs D, Hausen A, et al Value of urinary neopterin in the differential diagnosis of bacterial and viral infections. Klin Wochenschr. 1990;68(4):218–222. 2314009 10.1007/BF01662720

[59] Altmann DR, Jasperse B, Barkhof F, et al Sample sizes for brain atrophy outcomes in trials for secondary progressive multiple sclerosis. Neurology. 2009;72(7):595–601. 19005170 10.1212/01.wnl.0000335765.55346.fc PMC2818185

[60] Persson R, Lee S, Ulcickas Yood M, et al Infections in patients diagnosed with multiple sclerosis: A multi-database study. Mult Scler Relat Disord. 2020;41:101982. 32070858 10.1016/j.msard.2020.101982

[61] Devinney MJ, Wong MK, Wright MC, et al A role for blood–brain barrier dysfunction in delirium following non-cardiac surgery in older adults. Ann Neurol. 2023;94(6):1024–1103. 37615660 10.1002/ana.26771 PMC10841407

[62] Calvi A, Carrasco FP, Tur C, et al Association of slowly expanding lesions on MRI with disability in people with secondary progressive multiple sclerosis. Neurology. 2022;98(17):e1783–e1793. 35277438 10.1212/WNL.0000000000200144

[63] Elliott C, Wolinsky JS, Hauser SL, et al Slowly expanding/evolving lesions as a magnetic resonance imaging marker of chronic active multiple sclerosis lesions. Mult Scler J. 2019;25(14):1915–1925. 10.1177/1352458518814117 PMC6876256 30566027

